# Joint myocardial T_1_ and T_2_ mapping

**DOI:** 10.1186/1532-429X-17-S1-Q1

**Published:** 2015-02-03

**Authors:** Mehmet Akçakaya, Sebastian Weingärtner, Tamer A Basha, Sébastien Roujol, Reza Nezafat

**Affiliations:** 1 Medicine, Beth Israel Deaconess Medical Center, Harvard Medical School, Boston, MA, USA; 2 Computer Assisted Clinical Medicine, University Medical Center Mannheim, Heidelberg University, Mannheim, Germany

## Background

Recent studies suggest that quantitative myocardial T_1_ mapping allows assessment of focal and diffuse fibrosis in the myocardium [1]. Quantitative T_2_ mapping has also been proposed to overcome challenges associated with T_2_ weighted imaging [2]. These maps are traditionally acquired with different sequences, necessitating image registration to evaluate them jointly. A sequence that can jointly estimate T_1_ and T_2_ maps has been proposed [3], but it requires multiple relaxation cycles, which necessitates a lengthy free-breathing acquisition. In [4], an alternative joint estimation sequence was proposed based on the inversion-recovery SSFP curve. In this study, we sought to develop a saturation-recovery based heart-rate independent sequence that can be acquired in a breath-hold and that allows for simultaneous estimation of quantitative T_1_ and T_2_ maps.

## Methods

**Figure 1:**
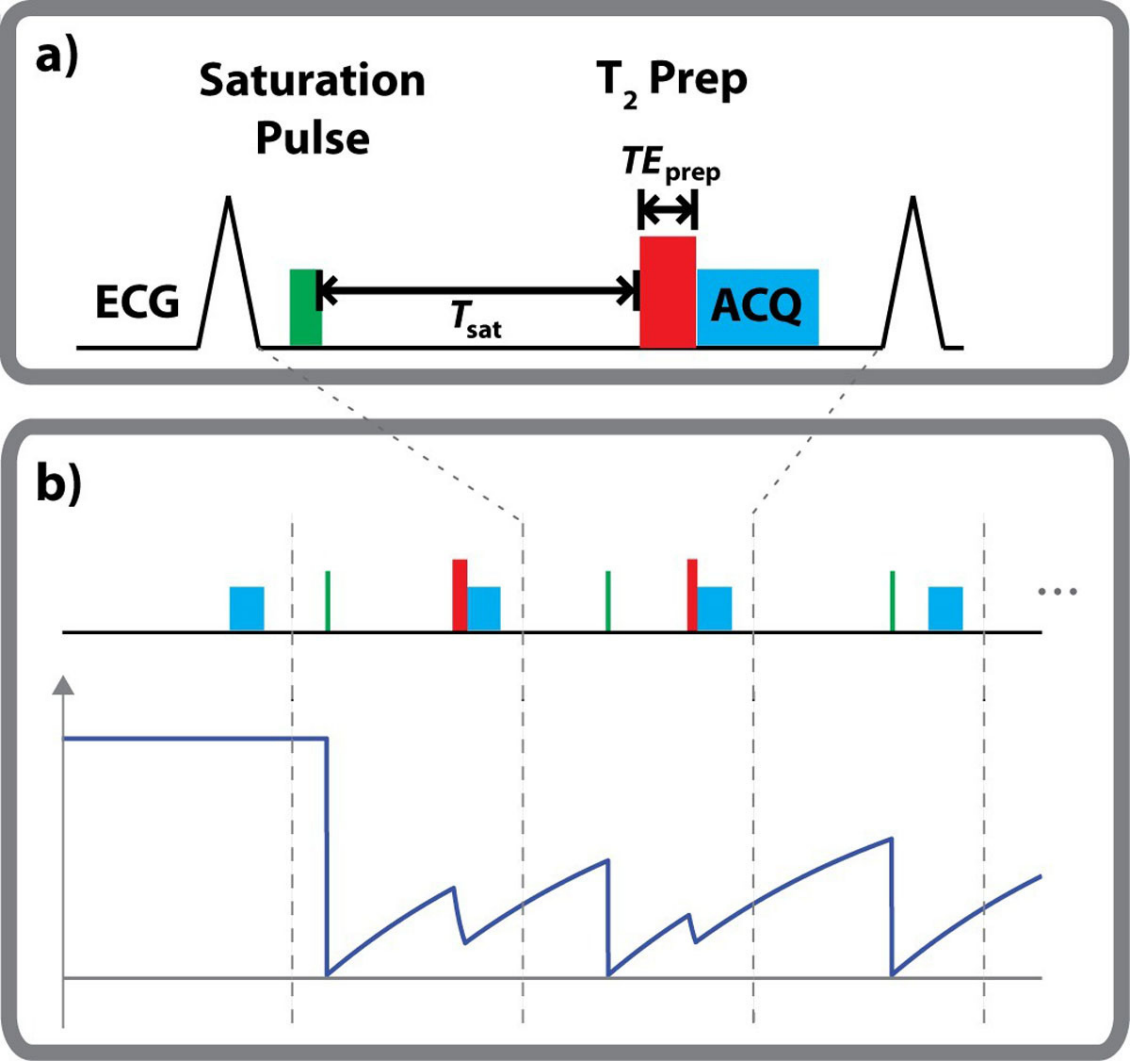
a) The sequence diagram for the proposed technique. A saturation pulse is applied in every R-R interval to eliminate the magnetization history. The longitudinal magnetization then recovers for T_sat_. Subsequently a T_2_-prep with echo length TE_prep_ is applied to generate the additional T_2_ weighting, after which a single shot SSFP image is acquired. b) The mapping sequence acquires the first image with no magnetization preparation (corresponding to T_sat_ = ∞ and TE_prep_ = 0), followed by 12 images (3 are shown) acquired with different T_sat_ and TE_prep_ values. The major characteristics of the longitudinal magnetization signal curve are depicted under the pulse sequence diagram.

The sequence diagram is depicted in Figure 1. At every heartbeat, a saturation pulse is applied to eliminate the magnetization history. The longitudinal magnetization then recovers for T_sat_ based on the T_1_ value. Subsequently a T_2_-prep pulse [5] with echo length TE_prep_ is applied to generate the additional T_2_ weighting, after which a single shot SSFP image is acquired. The process is repeated for 13 heartbeats with various (T_sat_^k^, TE_prep_^k^) corresponding to heartbeat *k*, to sample different T_1_-T_2_ weighted images. The first heartbeat is acquired with no magnetization preparation.

The T_1_ and T_2_ maps were estimated jointly by voxel-wise least squares fitting to a 4-parameter signal model, A (1- exp(-T_sat_^k^/T_1_)) exp(-TE_prep_^k^/T_2_) + B. Phantom imaging of 14 vials with different T_1_/T_2_ values were performed and compared to inversion-recovery and CPMG spin-echo references, respectively. Breath-held in-vivo imaging was performed on 5 healthy adult subjects, and the maps were compared to SASHA T_1_ maps [6] and to T_2_ maps [7].

## Results

**Figure 2:**
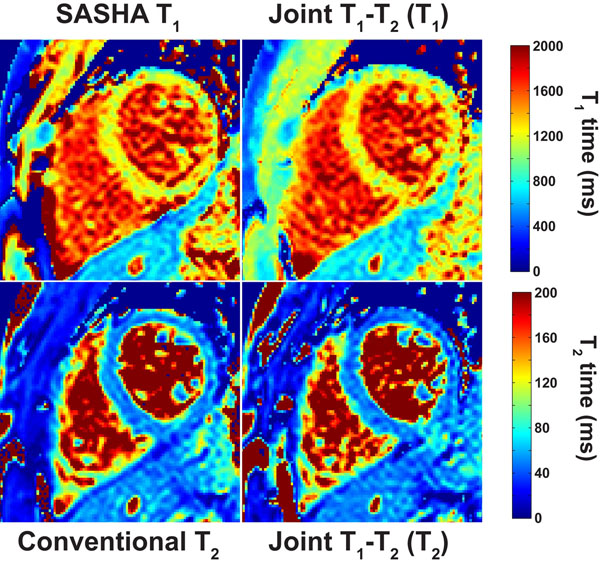
T_1_ and T maps from a healthy subject, acquired using the proposed technique, as well as SASHA T_1_ mapping, and conventional T_2_ mapping using 4 T_2_prep echo times. Both the T_1_ and T_2_ maps generated jointly with the proposed method are similar to the individual maps with similar magnetization preparations. The myocardial T_1_ and T_2_ values in the septum were 1211 ± 82 ms (SASHA T_1_), 1210 ± 92 ms (Joint T_1_-T_2_), 49.0 ± 5.8 ms (conventional T_2_) and 47.3 ± 6.5 ms (Joint T_1_-T_2_) for each technique. The methods generated with the proposed method were acquired in the same time as each individual map, and are jointly registered by design.

Phantom imaging resulted in T_1_ and T_2_ values not significantly different than the references (*P* = 0.481 and 0.479 respectively). Example in-vivo T_1_ and T_2_ maps are depicted in Figure 2, comparing various techniques. The T_1_ and T_2_ values were in good agreement (1211 ± 82 ms vs. 1210 ± 92 ms for T_1_; 49.0 ± 5.8 ms and 47.3 ± 6.5 ms for T_2_).

## Conclusions

The proposed sequence allows for the simultaneous estimation of accurate and jointly registered quantitative T_1_ and T_2_ maps with similar accuracy and precision to saturation-based T_1_ mapping and to T_2_ mapping of same duration.

## Funding

NIH:K99HL111410-01; R01EB008743-01A2.
